# Evaluation of the optimal cuff volume and cuff pressure of the revised laryngeal tube “LTS-D” in surgical patients

**DOI:** 10.1186/s12871-017-0308-4

**Published:** 2017-02-02

**Authors:** Marc Kriege, Christian Alflen, Johannes Eisel, Thomas Ott, Tim Piepho, Ruediger R. Noppens

**Affiliations:** 1 Department of Anesthesiology, University Medical Center of the Johannes Gutenberg-University Mainz, Mainz, Germany; 2 Department of Anesthesia & Perioperative Medicine, Western University; LHSC- University Hospital, 339 Windermere Road, London, ON N6A 5A5 Canada

**Keywords:** Airway management, Laryngeal tube, Cuff pressure, Ventilation

## Abstract

**Background:**

Recent case reports have indicated significant cuff overinflation when using the standard filling volume based on the manufacturer’s recommendations in older models of laryngeal tubes. The aim of this study was to determine the minimum cuff pressure needed to perform standardized ventilation without leakage in the new, revised model of the laryngeal tube “LTS-D”.

**Methods:**

After ethical approval, LTS-D was placed for ventilation in 60 anesthetized patients. The cuff was inflated to the recommended volume (#3: 60 ml, #4: 80 ml, and #5: 90 ml). After evaluation of the initial cuff pressure (CP), the CP was lowered in 10 cmH_2_O steps until a minimal cuff pressure of 30 cmH_2_O was achieved. The absence of an audible leak was required for a step-by-step reduction in the CP. Evacuated cuff volume, success rate, and airway injuries were documented. Data were expressed as medians (interquartile ranges [IQRs]). The comparison of CPs and cuff volumes was performed using the Mann-Whitney test.

**Results:**

After initial inflation, the CP ranged from 105 cmH_2_O [90–120; #5] to 120 cmH_2_O [110–120; #3]. Lowering the CP to 60 cmH_2_O resulted in a reduced cuff volume ranging from 47 ml [44–54; #3] to 77 ml [75–82; #5] compared to the initial inflation (*p* < 0.001). Leakage occurred more frequently when the CP was lowered to 40 cmH_2_O compared to the initial inflation (44/54 [81%]; *p* < 0.01). Using a CP between 50 cmH_2_O and 60 cmH_2_O, a leakage rate of 3/54 (5%) was observed, compared to a rate of 11/54 (21%) when using a CP lower than 50 cmH_2_O. The overall success rate was 90%, and airway injury occurred in 7% of patients (4/60).

**Conclusion:**

We found significant overinflation of the revised LTS-D using the recommended volume for initial cuff inflation. A CP of 60 cmH_2_O was found to be sufficient for ventilation in the majority of patients evaluated. Checking and adjusting the CP in laryngeal tubes is mandatory to avoid overinflation.

**Trial registration:**

ClinicalTrials.gov NCT02300337. Registered: 20 November 2014.

**Electronic supplementary material:**

The online version of this article (doi:10.1186/s12871-017-0308-4) contains supplementary material, which is available to authorized users.

## Background

**Fig. 1:**
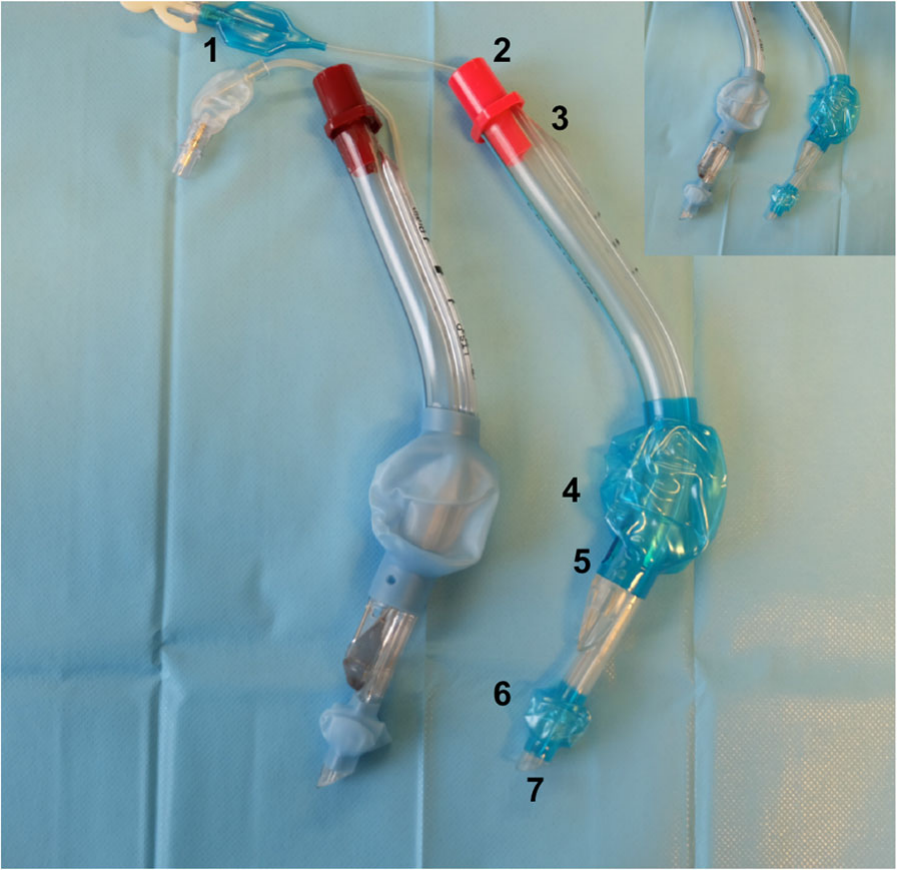
Old (*left*) and revised (*right*) LTS-D. 1: Pilot balloon, 2: color coded connector piece, 3: opening for gastric tube insertion, 4: pharyngeal balloon, 5: multiple ventilation outlets, 6: esophageal balloon, 7: esophageal opening

Since its introduction to the European market in 1999 and its approval by the FDA in 2003, the laryngeal tube (LT) has increasingly been used for airway management in anesthesia and emergency medicine [1–4]. From 1999 to today, several modifications have been made to the original version of the LT, including a softer tip and a high-volume low-pressure cuff designed to achieve maximum airway leak pressure and to avoid ischemic damage to the mucosa. The second-generation LT contains at the top of the ventilation orifices access for a gastric tube up to size 18 Fr. The LT can be inserted blindly without laryngoscopy and, after correct device insertion, the proximal cuff lies in the hypopharynx and the distal cuff is positioned in the upper esophagus. The revised LTS-D (S = Suction, D = disposable; VBM Medizintechnik GmbH, Sulz a. N., Germany) was introduced in 2015. It is now made from a softer material and uses a redesigned cuff (high volume, low pressure), and the distal cuff is more ovoid in shape (Fig. 1). Compared to the previous version of the LTS-D, which has a 45° curvature of the tube, the revised model has a 60° curvature, producing a more angulated tube that allows easier insertion into the pharynx. Additionally, the ventilation part of the revised LTS-D is now slimmer compared to the previous version. The LT has a 25 mm connector (color-coded for different sizes) and three black lines to indicate the correct depth of insertion. Several studies have reported complications associated with the previous versions of LT, such as a sore throat and blood traces on the cuff [5-7|. A correlation between complications with supraglottic airway devices and elevated cuff pressure (CP) is widely accepted [5–7]. Several authors have shown reduced pharyngeal mucosal microcirculation when the CP exceeds > 35 cmH_2_O [8–11].

In this prospective study, we aimed to determine the minimum CP for sufficient ventilation during general anesthesia with the new, revised LS-D. The secondary endpoints were the clinical performance of the LTS-D (success rate, time to insertion, minimum cuff volume, and postoperative airway morbidity).

## Methods

With the approval of the local Ethical Committee, Medical Association of the State of Rhineland-Palatinate, Germany (registry number: 837.176.14 [9415-F]), each potential participant was screened and written informed consent was obtained at least twelve hours before inclusion in this prospective, non-randomized clinical trial (ClinicalTrials.gov: NCT02300337, Registered: 20 November 2014).

### Patient selection

Patients underwent elective ophthalmic surgery under general anesthesia in a supine position at a tertiary university hospital.

Patients under 18 years old, with the risk of regurgitation, ASA classification IV and patients with an anticipated difficult airway were excluded from this study. Patients with a potential hazard for a failed laryngeal mask (e.g., edentulous/poor dentition, being male or having an elevated body mass index) were not excluded. Two anesthesiologists, both trained (>20 applications before the start of the trial) in placing LTs, performed placement in all cases.

### Setting and Intervention

The initial sizes of the new LTS-D were chosen in accordance with the manufacturer’s recommendation adapted for height (size 3: < 155 cm – 60 ml inflation volume; size 4: 155–180 cm – 80 ml; size 5: > 180 cm – 90 ml). Before each use, the cuff integrity was tested by full inflation and deflation to check for leaks. Both cuffs were lubricated with standard gel (Endosgel, Farco-Pharma GmbH, Köln, Germany). After three minutes of preoxygenation with a facemask, anesthesia was induced with sufentanil (0.2–0.3 μg.kg^−1^) and propofol (2–3 mg.kg^−1^), and anesthesia was maintained with either propofol infusion (TIVA) or volatile anesthetics. No muscle relaxants were used. The depth of anesthesia was controlled before insertion of LT by the loss of response to the jaw thrust maneuver. The LTS-D was introduced into the oropharynx against the hard palate and slid down until resistance was felt or the second bold black line on the LTS-D had passed between the upper and lower incisors. The cuff volume was inflated with a syringe using the recommended air volume (syringe color-coded by manufacturer) according to the size of the LT. After insertion, volume controlled ventilation (Primus/ Pallas, Dräger, Lübeck, Germany) was used with a tidal volume of 8 ml.kg^−1^ ideal body weight. The ventilator setting was standardized for each patient: fresh gas flow of 2 ltr.min^−1^, pressure limit 30 mbar, respiratory rate of 12 bpm, FiO_2_ 0.8, PEEP 0 mbar and a ratio of inspiration to expiration of 1:1. Leakage was defined as audible air escape during ventilation using the standardized ventilator settings.

### Outcomes measures

**Fig. 2:**
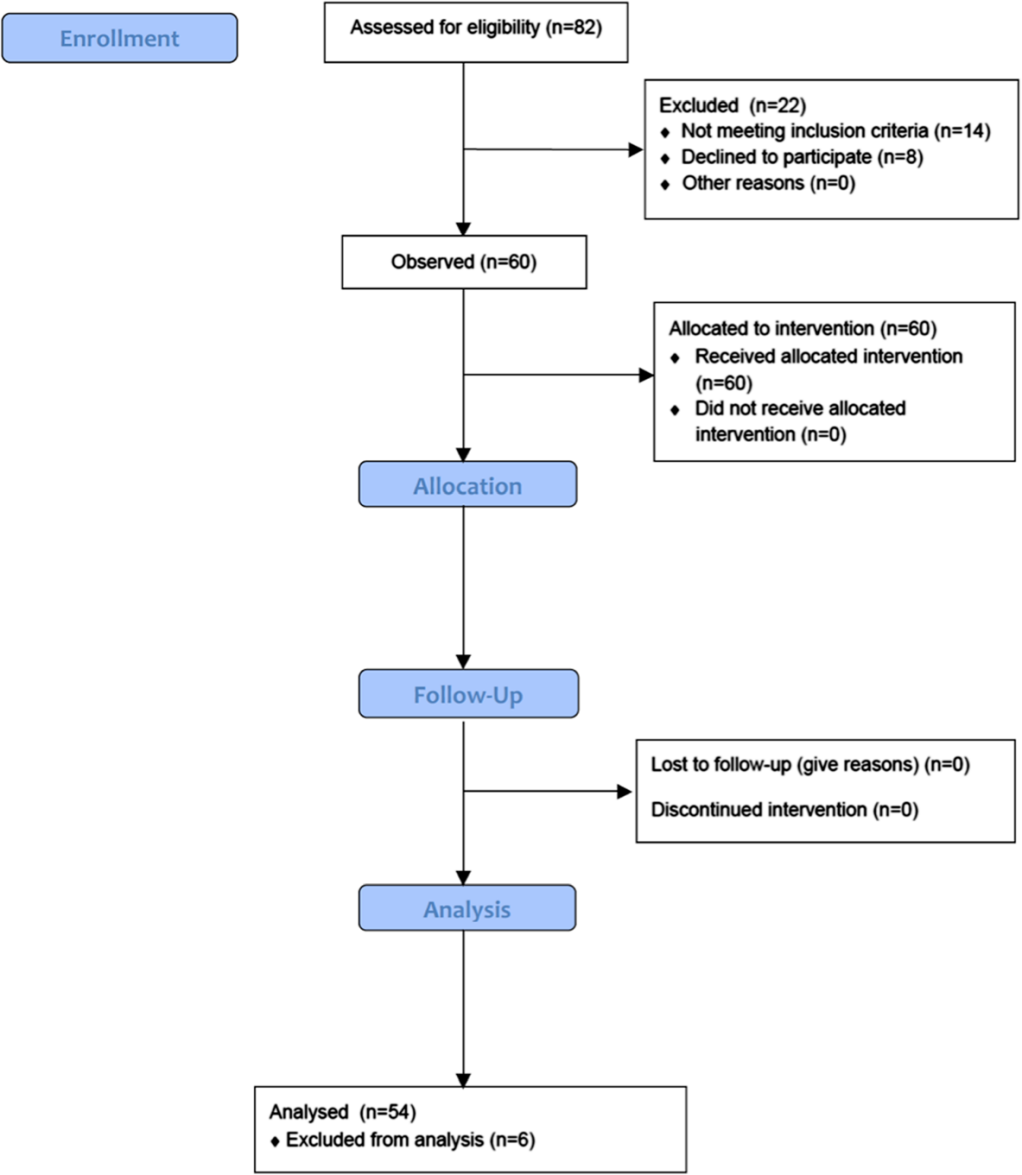
Study flow chart (CONSORT Flow Chart)

After placement, CP was controlled using a CP manometer (VBM CP gauge; VBM Medizintechnik, Sulz a. N, Germany). The CP manometer allows pressure readings up to 120 cmH_2_O, and consequently a CP above this limit was also recorded as 120 cmH_2_O. Initial values of CP, tidal volume (V_t_) and airway peak pressure were documented. After initial measurement, the CP was lowered to 60 cmH_2_O in the absence of an audible leakage. After recordings, CP was lowered in 10 cmH_2_O steps until a minimal CP of 30 cmH_2_O was achieved. We defined the lowest CP needed to perform standardized ventilation with a leakage rate lower than 10%. Between each step, a waiting time of 15 s was used to achieve stabilization before decreasing the CP. A unique set-up was used to reduce CP and we simultaneously measure the evacuated cuff volume using the manufacturer’s syringe, a three-way stopcock, and a CP manometer (Fig. 2). In the case of an audible leak, the cuff volume/CP was again increased until a seal was achieved. A CP without leakage was maintained at 30 cmH_2_O for the remainder of the anesthesia period.

A nurse measured the insertion time from the moment the mouth was opened until first ventilation. Successful insertion was defined as the ability to deliver adequate tidal volume and obtain a typical wave of CO_2_ on the capnometer. A maximum of two insertion attempts was allowed before failure of insertion was noted. The time for insertion was numbered only for the respective successful effort. In the case of failed placement, a laryngeal mask airway device or tracheal intubation was used to secure the airway. All evaluated data were documented using a standardized evaluation sheet. After the end of anesthesia, the device was removed and airway complications, such as blood staining on the cuffs, sore throat and hoarseness in the recovery room and 24 h later, were recorded.

### Statistical analysis

Sample size calculation: Based on similar studies [10–12], 60 patients were required to detect a local 1.7% level with > 90% power. Data analysis was performed using GraphPad Prism (Ver. 6.0 for MAC; GraphPad Software, San Diego, CA, USA). Data were expressed as the median (interquartile range [IQR]. Fisher’s exact test was used for comparison of successful ventilation at different CPs. The comparison of the various CPs and cuff volumes was performed with a Mann-Whitney test. A one-way analysis of variance (Kruskal-Wallis test) was used for multiple comparisons. The differences were considered statistically significant if the *p*-value was less than 0.05.

## Results

### Demographics

**Fig. 3:**
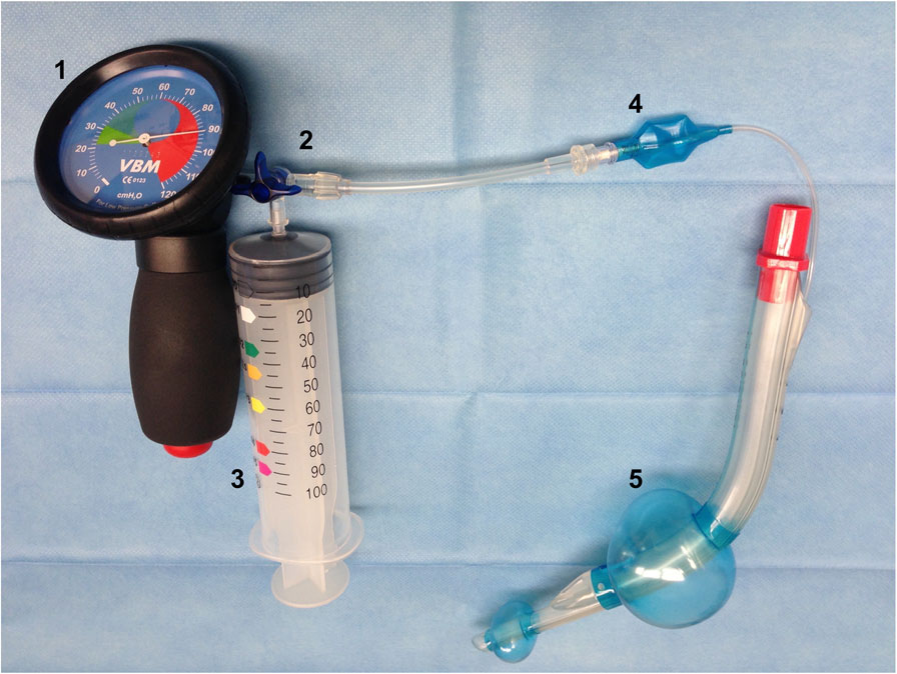
Study setting (revised LTS-D Size #4). A three-way-stop cock allowed simultaneous cuff pressure monitoring and volume removal by using the manufacturer syringe. 1: cuff pressure gauge, 2: three-way-stop cock, 3: color coded syringe for cuff inflation, 4: pilot baloon, 5: inflated pharyngeal- and asophageal cuff

From December 2014 to February 2015, 60 (33 female, 27 male) adult patients undergoing general anesthesia for ophthalmic surgery were included in this trial (Fig. 3). Patient characteristics are presented in Table 1.

**Table 1:** Demographic data and patients characters

Data	
Age (years)	68 [58.5–76]
Female/Male ratio	33/27
Height (cm)	168 [162–178]
BMI (kg/m)^2^	26 [23–28]
Mallampati class I/II/III	32/27/1
Thyromental distance (mm)	6 [5–6.5]
ASA score I/II/III	12/26/22
LTS-D Size 3/4/5	4/45/11
Surgical duration (min)	38 [21–62]
Propofol Induction doses (mg)	200 [150–220] (mean 2.5 mg.kg^−1^)
Sufentanil Induction doses (mcg)	10 [10–15] (mean 0.17 mcg.kg^−1^)

Data are given as median [IQR] or absolute numbers

### CP and air volume evacuation

The initial CP after successful placement (all sizes) was a median of 110 cmH_2_O [90–120], ranging from 105 cmH_2_O to 120 cmH_2_O (Table 2). Initial CPs were comparable between the three LTS-D sizes evaluated (Table 2).

**Table 2:** Cuff variables and ventilation variables for LTS-D

		LTS-D size			
		#3	#4	#5	*p*-value
After blocking with recommended volume	Cuff pressure [cmH_2_O]	120 [110–120]	110 [85–120]	105 [90–120]	0.35
	Cuff volume [ml]	60 [60–60]	80 [80–80]	90 [90–90]	<0.001
	Tidal volume [ml]	389 [366–559]	482 [449–559]	640 [613–672]	<0.001
	Peak pressure [cmH_2_O]	11 [9.8–16.5]	13 [12–16]	11.5 [11–13.3]	0.14
Cuff pressure controlled to 60 cmH_2_O	Cuff pressure [cmH_2_O]	60 [60–60]	60 [60–60]	60 [60–60]	1
	Cuff volume [ml]	47 [43.5–53.8]	70 [65–72.5]	77 [74.5–82]	<0.001
	Removed cuff volume [ml]	13 [6–17]	10 [8–15]	13 [8–16]	0.61
	Tidal volume [ml]	388 [364–424]	479 [443–532	656 [617–694]	<0.001
	Peak pressure [cmH_2_O]	12.5 [9.8–16.86]	13 [12–16]	11.5 [11–14]	0.26
Cuff pressure controlled to 50 cmH_2_O	Cuff pressure [cmH_2_O]	50 [50–50]	50 [50–50]	50 [50–50]	1
	Cuff volume [ml]	47 [28–54.6]	65 [60–70]	74 [68.5–78]	<0.001
	Removed cuff volume [ml]	13 [5–32]	15 [10–20]	16 [12–22]	0.49
	Tidal volume [ml	366 [312–397]	477 [442–518]	665 [627–702]	<0.001
	Peak pressure [cmH_2_O]	12.5 [9.8–16.8]	13 [11–16]	12 [11–14]	0.53
Cuff pressure controlled to 40 cmH_2_O	Cuff pressure [cmH_2_O]	40 [40–40]	40 [40–40]	40 [40–40]	1
	Cuff volume [ml]	49 [40–54]	60 [56–65]	68 [64–74]	<0.01
	Removed cuff volume [ml]	11 [6–20]	20 [15–25]	22 [17–27]	0.09
	Tidal volume [ml	352 [336–402]	467 [433–528]	649 [622–689]	<0.001
	Peak pressure [cmH_2_O]	13 [9–18]	13 [11–16]	12 [11–14.5]	0.72
Cuff pressure controlled to 30 cmH_2_O	Cuff pressure [cmH_2_O]	30 [30–30]	30 [30–30]	30 [30–30]	1
	Cuff volume [ml]	40 [35–45]	55 [52–60]	65 [55.8–67.3]	<0.01
	Removed cuff volume [ml]	20 [15–25]	25 [20–28]	25 [23–34]	0.15
	Tidal volume [ml]	347 [335–401]	460 [434–527]	644 [583–668]	<0.001
	Peak pressure [cmH_2_O]	13 [9–18]]	12 [11–15]	12 [10.3–14]	0.68

Data are given as median [IQR]

CPs were subsequently lowered to 30 cmH_2_O and resulted in a reduction of cuff volume of up to 25 ml (Table 2).

Tidal volume differed between LTS-D sizes because the ventilator setting was adjusted to the ideal body weight (Table 2). CP modifications did not influence the tidal volume.

With sufficient tidal volumes for ventilation, peak airway pressures ranged between 11 cmH_2_O and 13 cmH_2_O throughout the observation period (Table 2). The initial CP, the reduced CP of less than 60 cmH_2_O and variation of the evacuated cuff volume did not influence the peak pressures.

Using an uncontrolled CP, ventilation was possible in every patient without detectable leakage (Table 3). Leakage occurred at a CP of 60 cmH_2_O in two patients. Lowering of CP to 50 cmH_2_O resulted in an audible leak in three patients. Reducing the CP to under 50 cmH_2_O led to significant leakage, compared to an uncontrolled high CP (Table 3).

**Table 3:** Sufficient ventilation for different cuff pressures

			LTS-D size		
Cuff pressure	Sufficient ventilation [n]	*p*-value	#3	#4	#5
Uncontrolled	54 (100%)		4/4	40/40	10/10
60 cmH_2_O	52 (96%)	0.5	3/4	39/40	10/10
50 cmH_2_O	51 (94%)	0.24	3/4	39/40	9/10
40 cmH_2_O	44 (81%)	<0.01	3/4	33/40	8/10
30 cmH_2_O	35 (65%)	<0.001	2/4	28/40	5/10

Data are given as median [IQR] or absolute numbers

### Success rate and insertion time

The overall success rate with the LTS-D was 90% (54/60 patients overall; #3 4/4–#4 40/45–#5 10/11). Successful insertion after the first attempt was achieved in 51/60 (85%) and in an additional 3/60 (5%) at the second attempt. After failed placement using the LTS-D, a laryngeal mask airway was successfully placed in 5/6 patients (83%), and in one patient (17%) with an unexpected difficult airway, endotracheal intubation using a video laryngoscope was performed.

The time needed for successful placement at the first attempt was comparable between groups (all sizes: 23.5 s [19.75–32.75 s]; #3: 24 s [15–40 s]; #4: 24 s [20–33 s] - 22 s [14–28 s]; *p* = 0.6).

### Airway morbidity

The overall incidence of airway morbidity in this trial was 4/60 (7%). In all four patients, traces of blood were observed on the cuff after removal of the device. One of these four patients complained of a sore throat, hoarseness and dysphagia 30 min after surgery and 1 day later. Three additional patients complained of a sore throat and hoarseness the following day after surgery. No correlation was found between multiple attempts or failure of placement and the appearance of airway morbidity (e.g., sore throat; r = 0.25; *p* > 0.05).

## Discussion

In this prospective trial, we analyzed the optimal CP for ensuring ventilation without relevant leakage using the revised version of the LTS-D in surgical patients undergoing general anesthesia. According to the results of this trial, initial inflation to the manufacturer’s recommended cuff volume can be reduced by approximately 10 ml in all LTS-D sizes to achieve a CP of 60 cmH_2_O without compromising ventilation. A further reduction of CP below 50 cmH_2_O was associated with an increasing likelihood of leakage and cannot be recommended as an initial CP.

Patient demographic data were comparable to the data from other studies investigating the LTS-D in clinical settings [13–16].

In most patients, we could reduce the initial CP to 50 cmH_2_O and evacuated a cuff volume of approximately 15 ml. A higher CP than the mucosal perfusion pressure could induce tissue ischemia, which can promote the incidence of airway morbidity. Studies in living humans and cadavers have shown a direct correlation between the CP of laryngeal masks and mucosal injury [5, 8–11, 17]. The mucosal perfusion remains affected even if the CP is below 34 cmH_2_O (3). However, a further reduction of CP/cuff volume cannot be generally recommended since this has been associated with a high incidence of audible leakage of the LTS-D. Nevertheless, it is always advisable to check the CP in the LT and adjust it to an appropriate level before a leakage occurs to avoid potential mucosal injury.

The results on success rates are in agreement with published data comparing the previous versions of LTS or LTS-D with other supraglottic airway devices [15, 16, 18, 19]. In one study, an overall success rate of 70% was shown (3). Three other studies reported higher overall success rates of 96 and 98.5% [15, 16, 19]. The overall success rate was lower in our study, but comparable with a range of published data in clinical settings [16, 18, 19]. Data from out-of-hospital studies showed similar overall success rates ranging from 77 to 90% [20–22]. The time for successful insertion was approximately 24 s in this study and it verified other clinical studies, which showed insertion times from 14 s to 25 s [13–16, 19].

Unlike other studies, we limited the insertion attempts to two and set somewhat higher criteria (e.g., defined air leak) for successful insertion. To ensure a sufficient depth of anesthesia, a forced jaw thrust maneuver was performed in all patients. The absence of patient movement is generally accepted as a clinical indicator that depth of anesthesia is sufficient for supraglottic device insertion [23]. Performers of LTS-D had previous experience with the device in patients (>20) before starting this trial. The learning curve for successful insertion has been described as steep with 10 or fewer applications [24, 25].

The primary endpoint of this study was the evaluation of the lowest possible CP of the LTS-D in patients under general anesthesia without using a muscle relaxant. Several studies demonstrated a positive correlation between leak pressure and CP in supraglottic devices [26]. In laryngeal mask monitoring and adjusting CP to less than 40 cmH_2_O, the incidence and severity of a sore throat can be impressively reduced [27]. In this trial, the initial CP of LTS-D was 110 cmH_2_O [90–120] after insufflation using the recommended cuff volume (enclosed color-coded syringe). This finding is in agreement with another study, showing the initial CP in the previous version of the LTS-D size 4 exceeded 110 cmH_2_O in all cases. We found that evacuation of 11 ml [7.5–15] decreased the initial CP to 60 cmH_2_O.

Airway injury occurred in four patients (7%). In other studies, the incidence of complications (e.g., a mild sore throat and hoarseness) during recovery has been higher, ranging from 13% [13] to 24% [28]. Some authors confirmed that the incidence of airway morbidity in the previous version of the LTS-D was dependent on the combination of trauma on insertion (e.g., number of insertion attempts) and the pressure exerted by the cuff against the pharyngeal mucosa [24, 25].

### Study limitations

This study has several limitations. The sample size calculation was based on a study in pediatric patients. Up to the moment of planning this trial, no follow-up studies were found. Therefore, the estimated sample size might not be adequate. The study design was only powered for detecting CPs and not for detecting differences in airway morbidity.

Our findings showed no correlation between CP and airway morbidity, especially for sore throat and hoarseness. In contrast to other studies, we found no relationship between the number of attempts and a higher incidence of airway morbidity, although we limited the number of insertion attempts to two. Additionally, the LT was used for ventilation for a relatively short time of approximately 38 min in this trial. Our sample size was likely too small to analyze airway morbidity associated with the LT. We used an inspiration:expiration ratio of 1:1 because of this and the clinical standard in our institution for initially controlled ventilation when using supraglottic airway devices. A longer inspiratory time could allow lower insufflation pressure and could reduce the incidence of supraglottic device leaks. Additionally, these data do not allow conclusions to be drawn about the LTS-D CPs needed for sufficient ventilation during cardiopulmonary resuscitation with simultaneous chest compressions.

## Conclusions

In summary, the revised LTS-D can be successfully used for routine airway management in patients undergoing general anesthesia. However, these results revealed a significant overinflation of the revised LTS-D using the recommended volume for initial cuff inflation. Checking and adjusting the CP in laryngeal tubes is mandatory to avoid overinflation. Furthermore, the study demonstrated that cuff volumes can be reduced below the manufacturer’s recommendations without significantly influencing ventilation. However, a CP reduction below 50 cmH_2_O is associated with an increased risk of leakage. We recommend a 10 ml reduction in the volume for initial cuff inflation using the revised LTS-D and obligatory use of a CP manometer. Also, CP should be individualized for each patient to reduce the risk of injury.

## Additional file

Additional file 1: Minimal data set. (XLS 96 kb)

## Abbreviations

ASA: American Society of Anesthesiologists classification
BMI: Body Mass Index
C&L: Cormack and Lehane
CP: Cuff pressure
CO_2_: Carbon dioxide
LP: Leakage pressure
LTS-D: Laryngeal tube suction and disposable
PEEP: Positive end expiratory pressure
POGO: Percentage of glottic opening
TIVA: Total intravenous anesthesia
V_t_: Tidal volume

## References

[1] Genzwurker H, Finteis T, Hinkelbein J (2003). First clinical experiences with the new LTS. A laryngeal tube with an oesophageal drain. Anaesthesist.

[2] Mandal NG (2001). A new device has to be safe and reliable too. Anaesth.

[3] Agro F, Cataldo R, Alfano A, Galli B (1999). A new prototype for airway management in an emergency: the Laryngeal Tube. Resuscitation.

[4] Soar J, Nolan JP, Bottiger BW (2015). European Resuscitation Council Guidelines for Resuscitation 2015: Section 3. Adult advanced life support. Resuscitation.

[5] Ulrich-Pur H, Hrska F, Krafft P (2006). Comparison of mucosal pressures induced by cuffs of different airway devices. Anesthesiol.

[6] Burgard G, Mollhoff T, Prien T (1996). The effect of laryngeal mask cuff pressure on postoperative sore throat incidence. J Clin Anesth.

[7] Nott MR, Noble PD, Parmar M (1998). Reducing the incidence of sore throat with the laryngeal mask airway. Eur J Anaesthesiol.

[8] Keller C, Brimacombe J, Boehler M (2002). The influence of cuff volume and anatomic location on pharyngeal, esophageal, and tracheal mucosal pressures with the esophageal tracheal combitube. Anesthesiol.

[9] Keller C, Brimacombe J, Kleinsasser A (2003). Pharyngeal mucosal pressures with the laryngeal tube airway versus ProSeal laryngeal mask airway. Anasthesiol Intensivmed Notfallmed Schmerzther.

[10] Brimacombe J, Keller C, Puhringer F (1999). Pharyngeal mucosal pressure and perfusion: a fiberoptic evaluation of the posterior pharynx in anesthetized adult patients with a modified cuffed oropharyngeal airway. Anesthesiol.

[11] Eschertzhuber S, Brimacombe J, Kaufmann M (2012). Directly measured mucosal pressures produced by the i-gel and Laryngeal Mask Airway Supreme in paralysed anaesthetised patients. Anaesth.

[12] Licina A, Chambers NA, Hullett B (2008). Lower cuff pressures improve the seal of pediatric laryngeal mask airways. Paediatr Anaesth.

[13] Mihai R, Knottenbelt G, Cook TM (2007). Evaluation of the revised laryngeal tube suction: the laryngeal tube suction II in 100 patients. Br J Anaesth.

[14] Russo SG, Cremer S, Galli T, Eich C (2012). Randomized comparison of the i-gel, the LMA Supreme, and the Laryngeal Tube Suction-D using clinical and fibreoptic assessments in elective patients. BMC Anesthesiol.

[15] Thee C, Serocki G, Doerges V (2010). Laryngeal tube S II, laryngeal tube S disposable, Fastrach laryngeal mask and Fastrach laryngeal mask disposable during elective surgery: a randomized controlled comparison between reusable and disposable supraglottic airway devices. Eur J Anaesthesiol.

[16] Genzwuerker HV, Altmayer S, Hinkelbein J (2007). Prospective randomized comparison of the new Laryngeal Tube Suction LTS II and the LMA-ProSeal for elective surgical interventions. Acta Anaesthesiol Scand.

[17] Seet E, Yousaf F, Gupta S (2010). Use of manometry for laryngeal mask airway reduces postoperative pharyngolaryngeal adverse events: a prospective, randomized trial. Anesthesiol.

[18] Esa K, Azarinah I, Muhammad M (2011). A comparison between Laryngeal Tube Suction II Airway and Proseal Laryngeal Mask Airway in laparascopic surgery. Med J Malays.

[19] Cavus E, Deitmer W, Francksen H (2009). Laryngeal tube S II, ProSeal laryngeal mask, and EasyTube during elective surgery: a randomized controlled comparison with the endotracheal tube in nontrained professionals. Eur J Anaesthesiol.

[20] Kette F, Reffo I, Giordani G (2005). The use of laryngeal tube by nurses in out-of-hospital emergencies: preliminary experience. Resuscitation.

[21] Heuer JF, Barwing J, Eich C (2010). Initial ventilation through laryngeal tube instead of face mask in out-of-hospital cardiopulmonary arrest is effective and safe. Eur J Emerg Med.

[22] Maignan M, Koch FX, Kraemer M (2015). Impact of laryngeal tube use on chest compression fraction during out-of-hospital cardiac arrest. A prospective alternate month study. Resuscitation.

[23] Townsend R, Brimacombe J, Keller C (2009). Jaw thrust as a predictor of insertion conditions for the proseal laryngeal mask airway. Middle East J Anaesthesiol.

[24] Kurola J, Paakkonen H, Kettunen T (2011). Feasibility of written instructions in airway management training of laryngeal tube. Scand J Trauma Resusc Emerg Med.

[25] Langlois C, Pean D, Testa S (2007). The LTD laryngeal tube: a new single-use airway device. Ann Fr Anesth Reanim.

[26] Zhang L, Seet E, Mehta V (2011). Oropharyngeal leak pressure with the laryngeal mask airway Supreme at different intracuff pressures: a randomized controlled trial. Can J Anaesth.

[27] Wong JG, Heaney M, Chambers NA (2009). Impact of laryngeal mask airway cuff pressures on the incidence of sore throat in children. Paediatr Anaesth.

[28] Cattano D, Ferrario L, Patel CB (2012). Laryngeal Tube Suction-D, Combitube and Proseal Laryngeal Mask Airway: randomized clinical trial. J Anesthesiol Clin Sci.

